# Association between sleep problems and functional disability in community-dwelling older adults

**DOI:** 10.1186/s12877-024-04822-8

**Published:** 2024-03-13

**Authors:** Stefany Cristina Claudino Idalino, Letícia Martins Cândido, Katia Jakovljevic Pudla Wagner, Bruno de Souza Moreira, Alessandra de Carvalho Bastone, Ana Lúcia Danielewicz, Núbia Carelli Pereira de Avelar

**Affiliations:** 1 Laboratory of Aging, Resources and Rheumatology, Department of Health Sciences, Federal University of Santa Catarina, Campus Araranguá, Rod. Governador Jorge Lacerda, 3201, Urussanguinha, Araranguá, Santa Catarina 88906-072 Brazil; 2 Federal University of Santa Catarina, Campus Curitibanos, Rod. Ulysses Gaboardi, 300, Curitibanos, Santa Catarina 89520-000 Brazil; 3 Center for Studies in Public Health and Aging, Federal University of Minas Gerais and Oswaldo Cruz Foundation - Minas Gerais, Belo Horizonte, Minas Gerais Brazil; 4 Federal University of the Valleys of Jequitinhonha and Mucuri, Diamantina, Minas Gerais Brazil

**Keywords:** Sleep problems, Functional disability, Older adults

## Abstract

**Background:**

Sleep problems are frequently observed in older adults. They can lead to changes in the individual’s physical, occupational, cognitive, and social functioning, compromising the performance of activities of daily living and contributing to the occurrence of functional disability. This study evaluated the association between sleep problems and functional disability in community-dwelling older adults.

**Methods:**

This was a cross-sectional study with data from 10,507 Brazilian community-dwelling older adults participating in the 2013 National Health Survey. The exposure variable was self-reported sleep problems in the last two weeks. The outcome measure was functional disability assessed from self-reported questionnaires, categorized into basic activities of daily living (BADL) and instrumental activities of daily living (IADL), and defined as not being able to perform or having little or a lot of difficulty in at least one of the activities investigated in the domain of interest.

**Results:**

Older adults who reported sleep problems had 1.53 (95%CI: 1.34; 1.75) and 1.42 (95%CI: 1.26; 1.59) greater odds of having a disability in BADL and IADL when compared to individuals who reported having no sleep problems.

**Conclusions:**

Older adults with sleep problems were more likely to have a functional disability, both in BADL and IADL. Thus, it is important to implement strategies to screen for sleep problems in older adults in primary health care as a preventive strategy for functional disability.

## Background

Sleep problems can be defined as modifications that occur during sleep, which include insomnia, hypersomnia, circadian rhythm disorders, sleep breathing disorders, narcolepsy, and parasomnias [1]. The prevalence of these modifications has been high, ranging from 39.0 to 75.0% in community-dwelling older adults evaluated in Hong Kong [2] and 42.9% in middle-aged individuals in the United Kingdom [3]. In Brazil, data demonstrate that the prevalence of sleep problems in older adults varies between 32.7% and 44.9% [4–7]. These problems can lead to changes in the individual’s physical, occupational, cognitive, and social functioning, compromising the quality of life and the ability to perform activities of daily living (ADL) [8].

The ADL encompass tasks that are part of an individual’s daily life and are typically categorized into two levels: (1) basic (BADL), including mobility and personal self-care tasks [23, 24]; (2) instrumental (IADL), including more complex activities, such as occupational skills, financial management, shopping, transportation, utilization of technology (telephones and computers), and other household management responsibilities.

The disability to perform ADL (also known as functional disability) in older adults is associated with negative health outcomes, such as an increased risk of falls [9], mobility and balance deficits [10], dementia [11], mortality [12, 13], the incidence of cancers [13], frailty [14], longer response time in tasks [15] and attention deficit [16], as well as problems in short-term memory and performance level in practiced (word list learning) and non-practiced (grocery list and associative learning) memory tasks [17].

Contradictory findings about the relationship between sleep quality and functional abilities in older adults have been reported in the literature. Prior evidence suggests that sleep problems may predispose to functional disability [18–21] due to alterations in homeostatic balance. Such alterations can lead to repercussions on psychological function, immune system, and behavioral response, which could contribute to functional disability [22]. On the other hand, Pereira et al. [18] and Okoye et al. [19] found no association between self-reported sleep problems and functional disability in BADL and IADL in older adults. Considering the current body of knowledge, additional research is necessary to generate more robust conclusions on the topic. Investigating the association between these conditions in older adults can be useful for implementing screening strategies for sleep problems in this population, thus preventing their consequences. Therefore, the objective of the present study was to examine the association between self-reported sleep problems and functional disability in BADL and IADL in Brazilian community-dwelling older adults. We hypothesized that sleep problems can be considered as a potential contributor or marker of physical function limitations and disability in older adults.

## Methods

### Study design and population

This was a cross-sectional study carried out with data from the National Health Survey (PNS acronym, in Portuguese), collected in 2013 (IBGE, 2014) [25]. The PNS [26] is a nationwide, household-based survey, carried out in partnership with the Ministry of Health, Oswaldo Cruz Foundation, and the Brazilian Institute of Geography and Statistics (IBGE acronym, in Portuguese). For the dissemination of the research, a PNS website (www.pns.fiocruz.br) was created, containing the preparatory phase of the research, the theoretical framework, the objectives, the sampling plan, the questionnaires, the interview and physical measurement handbooks, and the ethics approval document (approved by the National Research Ethics Committee of the National Health Council, under process nº 328.159, on June 26, 2013). The informed consents for the research were obtained in two stages. In the first stage, informed consent for the household interview was obtained from the household informant before the commencement of data collection. In this process, the information collection agent read the Informed Consent Form to the individual, who signed it if they agreed to participate in the research. In the second stage, informed consent was sought from the selected adult resident (≥ 18 years) for the individual interview. In this case, consent was requested for each of the procedures separately: conducting the interview; performing anthropometric and blood pressure measurements; and collecting blood and urine from those included in the sample selected for laboratory tests. Before the individual interview, the information collection agent explained to the interviewee, clearly and objectively, the voluntary nature of their participation and the possibility to (i) refuse to answer any question, (ii) interrupt the interview at any time, or (iii) opt out of certain planned measurements and laboratory tests, even after giving prior consent. All Informed Consent Forms are available on the PNS website (https://www.pns.icict.fiocruz.br/aspectos-eticos/).

The IBGE is main Brazil’s provider of data and information and offers a complete and current view of the country’s population, through the performance of its main functions that include, but are not limited to, the production and analysis of statistical information, consolidation of geographic data, and documentation and dissemination of information.

The PNS is part of the Integrated System of Household Surveys, also developed by the IBGE and had its first survey in 2013, and was repeated in 2019 [27]. This nationwide survey aimed to collect information about the health conditions of the population, surveillance of chronic non-communicable diseases and their risk factors, and performance of the national health system concerning access and use of available services and continuity of care.

The target population for this research comprises residents of private households in Brazil, excluding those located in special census sectors (such as barracks, military bases, accommodations, camps, vessels, penitentiaries, penal colonies, prisons, jails, nursing homes, orphanages, convents, and hospitals). The following were considered as losses: closed or vacant households, residents’ refusal to participate in the interview, and failure to interview the informant after three or more attempts, even with scheduled visitations. Then, within each household, a resident aged 18 years and over was randomly selected, based on the list of residents obtained at the time of the interview. In the current study, only participants aged 60 years and over were eligible for the analyses.

### Sampling and data collection procedures

The PNS sampling plan was designed in three stages: the primary units were the census tracts or set of tracts, the secondary units were the households, and the tertiary units were adult residents (≥ 18 years).

The selection of the subsample was performed using simple random sampling, with 6,069 census tracts and 81,767 households being sampled. Considering a 22% non-response rate, 62,986 households in the country received interviewers. Concerning individuals, the sample proportion varied according to the indicator of interest. The database provided by the IBGE presents data on 205,546 individuals aged 18 years and over, and of these, 11,177 older adults (aged 60 years and over) who responded to the questionnaire on sleep problems and functional disability. Of these, 670 older adults were excluded due to a lack of information on some variables of interest in this study, resulting in a sample of 10,507 participants.

### Exposure variable

The exposure variable was the self-report of sleep problems present in module N of the PNS questionnaire (question N010), evaluated through the following question: “*In the last two weeks, how often did you have sleep problems, such as difficulty falling asleep, waking up frequently at night, or sleeping more than usual?”*. The response options were: (1) none day; (2) less than half of the days; (3) more than half of the days; (4) almost every day. This question was categorized into: no history of sleep problems (answer 1) and with a history of sleep problems (answers 2, 3, and 4).

### Outcome variables

The analyzed outcomes were obtained with the question “*In general, what degree of difficulty do you have for…?*”. Six BADL (getting in/out of bed, eating, walking on a flat surface, showering, dressing, and going to the bathroom) and nine IADL (taking care of appearance, climbing a flight of stairs, taking medication, walking close to home, shopping, preparing meals, cutting toenails, taking the bus/taxi, and cleaning the house) were investigated. The questions were obtained from module K of the PNS questionnaire. The response options were: (1) do not perform; (2) have great difficulty; (3) have little difficulty; (4) have no difficulty. Older adults who reported not being able to perform, or having little or great difficulty in at least one of the activities investigated in each domain of interest were classified as having a functional disability.

### Adjustment variables

The adjustment variables used in this study were sex (male; female) [28], age group (60–69, 70–79 or ≥ 80 years) [29], schooling (no formal education; 1–4; 5–8; 9–11; ≥12 years) [30], total number of chronic diseases (0; 1–2; ≥3) [31], self-report of the physical activity practice [In the last three months, have you practiced any type of physical exercise or sport? (no; yes)] [32], intellectual disability [Do you have an intellectual disability? (no; yes)], self-reported medical diagnosis of depression (no; yes), self-rated health [In general, how do you rate your health? (very good/good; regular; bad/very bad)], adequate consumption of fruits and vegetables [recommended consumption at least 25 times a week, with minimum consumption of five fruits (including juice) and five vegetables a day (no, considering those who do not consume these foods and those who do not consume the adequate amount; yes)], and alcohol consumption [How often do you usually consume any alcoholic beverage? (I never drink; less than once a month; once or more a month)].

### Statistical analysis

The Stata software (Stata Corp., College Station, Texas, USA), version 15.0, was used. All analyses considered the effect of the study design, incorporating the sample weights using the *svy* command. Descriptive analyses were performed for sleep problems and all adjustment variables for the total sample and according to functional disability domains. Logistic regression analyses were conducted to investigate the association between self-reported sleep problems and functional disability in BADL or IADL, estimating the crude and adjusted *odds ratios* (OR) and their respective 95% confidence interval (95%CI).

## Results

Data from 10,507 older adults were analyzed. There was a predominance of females (61.2%) and participants aged between 60 and 69 years (56.0%). The sociodemographic and lifestyle characteristics of the evaluated older adults are described in Table 1. The prevalence rates of sleep problems and functional disability in BADL and IADL in the total sample were 34.7% (95%CI: 33.7; 35.7), 15.6% (95%CI: 14.9; 16.4), and 28.7% (95%CI: 27.8; 29.6), respectively. Among older adults with sleep problems, 23.0% (95%CI: 21.6; 24.6) and 38.0% (95%CI: 36.3; 39.7) had a functional disability in BADL and IADL, respectively (Table 1).

**Table 1:** Sleep problems, sociodemographic and lifestyle characteristics of Brazilian older adults according to functional disability in basic (BADL) and instrumental (IADL) activities of daily living. National Health Survey (PNS), Brazil, 2013

Variables	Total [*n* = 10,507]		BADL disability [*n* = 1,664]	IADL disability [*n* = 3,109]
	N	% (95%CI)	% (95%CI)	% (95%CI)
**Sleep problems**				
No	6,867	65.3 (64.2; 66.2)	11.7 (10.9; 12.5)	23.8 (22.7; 24.9)
Yes	3,640	34.7 (33.7; 35.7)	23.0 (21.6; 24.6)	38.0 (36.3; 39.7)
**Sex**				
Male	4,132	38.9 (37.7; 39.7)	14.0 (12.9; 15.1)	23.3 (22.0; 24.7)
Female	6,375	61.2 (60.2; 62.2)	16.6 (15.7; 17.6)	32.1 (30.9; 33.3)
**Age group**				
60–69 years	5,845	56.0 (54.9; 57.0)	10.3 (9.5; 11.1)	17.0 (16.0; 18.0)
70–79 years	3,248	30.7 (29.6; 31.5)	16.8 (15.5; 18.2)	34.4 (32.7; 36.1)
≥ 80 years	1,414	13.3 (12.7; 14.0)	35.0 (32.4; 37.7)	64.7 (62.1; 67.2)
**Schooling**				
No formal education	3,237	28.4 (27.5; 29.4)	22.7 (21.2; 24.4)	45.2 (43.4; 47.1)
1–4 years	3,363	33.0 (31.9; 34.0)	15.3 (14.1; 16.7)	28.5 (26.9; 30.1)
5–8 years	1,257	12.1 (11.4; 12.8)	14.1 (12.2; 16.2)	22.5 (20.1; 25.0)
9–11 years	1,487	14.5 (13.7; 15.2)	9.9 (8.4; 11.7)	15.7 (13.9; 17.7)
≥ 12 years	1,163	12.0 (11.1; 12.6)	7.7 (6.2; 9.4)	12.1 (10.3; 14.2)
**Total number of chronic diseases**				
0	2,582	24.0 (23.0; 24.8)	6.8 (5.8; 7.9)	16.5 (15.0; 18.0)
1–2	5,781	54.6 (53.5; 55.6)	14.1 (13.2; 15.1)	28.2 (27.0; 29.4)
≥ 3	2,144	21.4 (20.5; 22.3)	29.2 (27.2; 31.3)	43.7 (41.5; 45.9)
**Physical activity practice**				
No	8,210	76.8 (75.8; 77.6)	18.2 (17.3; 19.1)	33.1 (32.0; 34.2)
Yes	2,297	23.2 (22.2; 24.1)	6.9 (5.9; 8.0)	14.2 (12.8; 15.8)
**Intellectual disability**				
No	10,426	99.2 (99.0; 99.4)	15.2 (14.4; 15.9)	28.2 (27.2; 29.1)
Yes	81	0.8 (0.5; 0.9)	71.5 (58.3; 81.8)	96.8 (88.9; 99.1)
**Self-reported medical diagnosis of depression**				
No	9,570	90.3 (89.6; 90.9)	15.0 (14.2; 15.8)	27.6 (26.6; 28.5)
Yes	937	9.7 (9.0; 10.3)	21.7 (19.0; 24.6)	39.4 (36.2; 42.7)
**Self-rated health**				
Very good/good	4,634	45.6 (44.5; 46.6)	6.1 (5.4; 6.8)	15.4 (14.3; 16.5)
Regular	4,506	42.1 (41.0; 43.1)	18.4 (17.2; 19.6)	34.2 (32.7; 35.7)
Bad/very bad	1,367	12.3 (11.6; 12.9)	41.4 (38.7; 43.3)	59.2 (56.5; 61.9)
**Adequate consumption of fruits and vegetables**				
No	8,579	80.0 (79.1; 80.8)	16.4 (15.5; 17.3)	30.0 (29.0; 31.0)
Yes	1,928	20.0 (19.1; 20.8)	12.4 (10.9; 14.0)	23.4 (21.5; 25.4)
**Alcohol consumption**				
I never drink	8,258	77.3 (76.4; 78.1)	17.7 (16.8; 18.6)	32.8 (31.7; 33.9)
Less than once a month	917	9.1 (8.5; 9.7)	9.3 (7.5; 11.4)	17.7 (15.2; 20.4)
Once or more a month	1,332	13.6 (12.8; 14.2)	7.9 (6.5; 9.5)	12.7 (10.9; 14.7)

All estimates took into account the weights of the individuals and the complex sample design

95%CI: 95% confidence interval

The adjusted analysis showed that older adults with sleep problems presented 1.53 (95%CI: 1.34; 1.75) and 1.42 (95%CI: 1.26; 1.59) greater odds of having a functional disability in BADL and IADL, respectively, when compared to older adults who reported no sleep problems (Table 2).

**Table 2:** Crude and adjusted associations between the presence of sleep problems and functional disability in basic (BADL) and instrumental (IADL) activities of daily living among Brazilian older adults. National Health Survey (PNS), Brazil, 2013

Sleep problems	BADL disability		IADL disability	
	Crude	Adjusted#	Crude	Adjusted#
	OR (95%CI)	OR (95%CI)	OR (95%CI)	OR (95%CI)
No	1.00	1.00	1.00	1.00
Yes	**2.26 (2.01; 2.54)**	**1.53 (1.34; 1.75)**	**1.96 (1.79; 2.16)**	**1.42 (1.26; 1.59)**

All estimates took into account the weights of the individuals and the complex sample design

#Adjusted for sex, age group, schooling, total number of chronic diseases, physical activity practice, intellectual disability, self-reported medical diagnosis of depression, self-rated health, adequate consumption of fruits and vegetables, and alcohol consumption. OR: odds ratio; 95%CI: 95% confidence interval

*Note* Values in bold indicate a statistically significant association

## Discussion

The results of the present study showed that older adults with sleep problems were more likely to have a functional disability in BADL and IADL when compared to older adults without sleep problems. Similar results were observed in the studies by Dam et al. [20] and Goldman et al. [21], in which American older adults with sleep problems objectively assessed using polysomnography and actigraphy, respectively, had lower values of grip strength and walking speed, as well as a disability to get up from a chair without assistance and to complete a walking path, suggesting that interrupted sleep at night results in worse physical and functional performance. These findings were also found by Liu et al. [33] who demonstrated that both short (≤ 5 h/day) and long (> 9 h/day) sleep duration were predictive of disability in IADL among Chinese middle-aged and older adults.

The association between sleep problems and functional disability can be explained by several physiological mechanisms [34–37]. First, the decline in sleep quality has negative repercussions on vitality and attention, which can predispose the individual to depression [36]. In addition, short sleep duration can cause adverse effects on cognitive performance, such as longer reaction times, impaired executive function related to the prefrontal cortex, and working memory skills [35]. On the other hand, prolonged sleep (hypersomnia) is linked to higher rates of gray matter atrophy in frontotemporal areas among older adults, which can potentially impair memory [38, 39]. All of these cognitive and mood alterations are associated with functional disability [40].

Another possible mechanism linking sleep problems with a functional disability was recently proposed by Liu et al. [37]. These authors investigated the association of sleep duration and efficiency with plasma levels of beta-amyloid protein (Aβ) and found that there is an increase in Aβ load in cerebrospinal fluid in situations of deprivation or low efficiency of sleep, concluding that the excitotoxic effects and neuroinflammatory properties of Aβ seem to play an important role in inducing neurodegeneration. It is known that neurodegeneration compromises the regeneration of muscle fibers, predominantly type II fibers [41, 42], reducing the ability to produce muscle strength, which would be directly related to functional disability.

In addition to the role of Aβ protein, the action of inflammatory markers may explain the association between sleep problems and functional disability [45, 44]. Kuo et al. [45] found that older adults with high levels of C-reactive protein and high albumin/creatinine ratio had 1.60 (95%CI: 1.13; 2.28) and 1.71 (95%CI: 1.20; 2.45) greater odds of having a functional disability in BADL, respectively. Furthermore, an association was found between short sleep duration and high levels of high-sensitivity C-reactive protein (hs-CRP) in healthy adults [44]. Elevated levels of hs-CRP have been linked to inflammation, aging, sarcopenia, cancer, angina pectoris, coronary artery disease, chronic obstructive pulmonary disease, stroke, and increased risk of mortality [44]. Therefore, it is plausible to infer that the short sleep duration would lead to an increase in C-reactive protein levels, which in turn would increase the chances of the individual developing functional disability.

Although the aforementioned studies have demonstrated the association between sleep problems and functional disability, it is possible to observe controversies in this association, especially due to differences in the assessment manner (subjective or objective) of sleep problems. Pereira et al. [18] found no association between self-reported sleep problems and functional disability in BADL and IADL in a random sample of 388 older adults from three Basic Health Units of Teresina, Piauí State, Brazil. Okoye et al. [19] also showed that subjective complaints of sleep problems were not significantly associated with disability in BADL/IADL in American older adults. However, objective measures of prolonged sleep and easier awakening from sleep were associated with worse physical function [19]. The authors pointed out that the lack of association between subjectively assessed sleep problems and disability may have occurred because the subjective measurement was limited to a single item of a depression screening tool (Item 3 of the Patient Health Questionnaire-9 - PHQ-9) [19].

In addition to the physiological mechanisms suggested in the association between sleep problems and functional disability, other factors such as predisposing, precipitating, and perpetuating ones may be associated with sleep problems [45]. Predisposing factors include sociodemographic characteristics, such as sex [28, 46], age [29], low education or income levels [30], and psychological characteristics, such as divorce or the death of a spouse. In addition, lifestyle habits, such as smoking and alcoholism [47] also affect sleep. Precipitating factors include stressful life events or medical conditions that can disrupt sleep [48], such as respiratory symptoms and physical disability [49]. Prior research has also shown that patients with depression and generalized anxiety disorder have higher insomnia rates [50]. In turn, perpetuating factors include spending excessive time in bed, frequent naps, and conditioning (increased anxiety before sleep onset due to fear of going another sleepless night) [48].

Several treatments can be indicated for sleep problems. An interesting option would be non-pharmacological management since it is not susceptible to residual side effects such as those commonly observed with pharmacological treatment involving the use of benzodiazepines and other sleep-inducing drugs [51]. Regular physical exercise is a simple strategy suggested to deal with sleep problems in older adults because it can promote relaxation and increase core body temperature, thus facilitating sleep initiation and maintenance [52]. In addition to physical exercise, the adoption of some behaviors, such as avoiding the use of electronic devices before going to sleep, can help older adults obtain better sleep quality, as exposure to screen light inhibits the production of serum melatonin by the pineal gland, which directly influences the biological clock and consequently increases the occurrence of sleep problems [6, 53].

Some limitations of the present study should be highlighted. The variables related to sleep problems and functional disability were obtained by self-report, thus making it possible for some types of bias to occur, such as memory and social desirability. Due to the cross-sectional nature of this study, the temporality between exposure and outcome variables and the causality of the associations cannot be established and reverse causality is a possibility. However, it should be highlighted that subjective measures allow greater applicability in population-based studies, as well as their use in clinical settings. Furthermore, other important variables related to sleep, including the presence of insomnia, total sleep time, and daytime sleepiness were not collected in the PNS, which prevents a deeper understanding of the relationship between sleep problems and functional disability in the older population. It is also known that pain is an important factor for disability and sleep problems, and may mediate the association between these variables [54]. However, the PNS did not investigate pain in the 2013 questionnaire. Thus, our findings need to be interpreted with caution in light of these limitations.

Among the strengths of this study, it is noteworthy that our work was carried out using data from a nationwide survey conducted in the largest country in Latin America. Furthermore, to our knowledge, this was the first study to investigate the association between sleep problems and functional disability in a nationwide sample of older adults. These findings provide support for the implementation of screening strategies for this condition to prevent or delay the occurrence of functional disability in BADL and IADL in community-dwelling older adults. An example of a screening strategy that could be easily used in primary health care would be the Consensus Sleep Diary [55], an accessible resource, that includes detailed questions to evaluate the sleep history, such as sleep onset latency, number and duration of awakenings, wakefulness after sleep onset, total sleep time, as well as qualitative aspects, such as subjective sleep quality and sleep satisfaction.

## Conclusion

In the population studied, older adults with sleep problems were more likely to have a functional disability, both in BADL and IADL. Thus, it becomes important to implement screening strategies for sleep problems in primary health care as a preventive strategy for functional disability among Brazilian community-dwelling older adults. Future studies investigating the relationship between sleep problems and functional disability should be conducted in older populations of different nationalities to establish more robust conclusions on this topic.

## Data Availability

The datasets used and/or analyzed during the current study are available from the corresponding author upon reasonable request.
